# Supplementary material for: On the objectivity, reliability, and validity of deep learning enabled bioimage analyses
Source: eLife. 2020 Oct 19;9:e59780. doi: 10.7554/eLife.59780 (PMC7710359; doi:10.7554/eLife.59780)
Supplement: Figure 5—source data 2. [file elife-59780-fig5-data2.docx]

| **laboratory** | **# total images** | **# training/ validation images** | **# test images** | **imaging**  **technique** | **resolution** | **pixel dimensions** | **model organism** | **fluorescent feature** |
| --- | --- | --- | --- | --- | --- | --- | --- | --- |
| *Lab-Mue* | 29 | 5 | 0 | confocal LSM | 0.8 px / µm | 1024 x 1024 | Mus musculus | cFOS (nuclear) |
| *Lab-Inns1* | 24 | 5 | 0 | epifluorescence microscopy | 1 px / µm | 1344 x 1024 | Mus musculus | cFOS (nuclear) |
| *Lab-Inns2* | 30 | 5 | 0 | epifluorescence microscopy | 2 px / µm | 1376 x 1038 | Mus musculus | cFOS (nuclear) |
| *Lab-Wue1* | 319 | 36 | 9 | confocal LSM | 1.61 px / µm | 1024 x 1024 | Mus musculus | cFOS (nuclear) |
| *Lab-Wue2* | 40 | 5 | 0 | confocal LSM | 12.87 px / µm | 1024 x 1024 | Danio rerio | GABA (somatic) |
