# Supplementary material for: On the objectivity, reliability, and validity of deep learning enabled bioimage analyses
Source: eLife. 2020 Oct 19;9:e59780. doi: 10.7554/eLife.59780 (PMC7710359; doi:10.7554/eLife.59780)
Supplement: Transparent reporting form [file elife-59780-transrepform.docx]

***eLife’s* transparent reporting form**

We encourage authors to provide detailed information *within their submission* to facilitate the interpretation and replication of experiments. Authors can upload supporting documentation to indicate the use of appropriate reporting guidelines for health-related research (see [EQUATOR Network](http://www.equator-network.org/%20)), life science research (see the [BioSharing Information Resource](https://biosharing.org/)), or the [ARRIVE guidelines](http://www.plosbiology.org/article/info:doi/10.1371/journal.pbio.1000412) for reporting work involving animal research. Where applicable, authors should refer to any relevant reporting standards documents in this form.

If you have any questions, please consult our Journal Policies and/or contact us: [editorial@elifesciences.org](mailto:editorial@elifesciences.org).

**Sample-size estimation**

- You should state whether an appropriate sample size was computed when the study was being designed
- You should state the statistical method of sample size computation and any required assumptions
- If no explicit power analysis was used, you should describe how you decided what sample (replicate) size (number) to use

Please outline where this information can be found within the submission (e.g., sections or figure legends), or explain why this information doesn’t apply to your submission:

We did not implement any statistical approach to a priori define the sample sizes. Our sample sizes correspond to sample sizes that are generally used in this field of research and based on our previous experience with similar experiments or those that have been routinely used in studies applying analogous methodological approaches and published in the field of neuroscience.

**Replicates**

- You should report how often each experiment was performed
- You should include a definition of biological versus technical replication
- The data obtained should be provided and sufficient information should be provided to indicate the number of independent biological and/or technical replicates
- If you encountered any outliers, you should describe how these were handled
- Criteria for exclusion/inclusion of data should be clearly stated
- High-throughput sequence data should be uploaded before submission, with a private link for reviewers provided (these are available from both GEO and ArrayExpress)

Please outline where this information can be found within the submission (e.g., sections or figure legends), or explain why this information doesn’t apply to your submission:

Our bioimage datasets contain only biological replicates, no technical replicates. The numbers of both analyzed animals (N) and analyzed images (n) for each bioimage analysis are given in the figure legends. Likewise, the number of deep-learning models or ensembles that were used for the analyses are given in each figure legend. We provide our data and the respective analysis source code as described in the section “Data and software availability”. Outlier testing is described in the section "Statistical analysis of fluorescent feature quantifications".

**Statistical reporting**

- Statistical analysis methods should be described and justified
- Raw data should be presented in figures whenever informative to do so (typically when N per group is less than 10)
- For each experiment, you should identify the statistical tests used, exact values of N, definitions of center, methods of multiple test correction, and dispersion and precision measures (e.g., mean, median, SD, SEM, confidence intervals; and, for the major substantive results, a measure of effect size (e.g., Pearson's r, Cohen's d)
- Report exact p-values wherever possible alongside the summary statistics and 95% confidence intervals. These should be reported for all key questions and not only when the p-value is less than 0.05.

Please outline where this information can be found within the submission (e.g., sections or figure legends), or explain why this information doesn’t apply to your submission:

The general statistical analysis is described in the section "Statistical analysis".

Detailed information for each statistical analysis (e.g. statistical test, test statistic, N- /

n-numbers, and p-values) are either described in the corresponding figure legends wherever possible or is provided in a source data file which is referenced in the respective figure legend.

(For large datasets, or papers with a very large number of statistical tests, you may upload a single table file with tests, Ns, etc., with reference to sections in the manuscript.)

**Group allocation**

- Indicate how samples were allocated into experimental groups (in the case of clinical studies, please specify allocation to treatment method); if randomization was used, please also state if restricted randomization was applied
- Indicate if masking was used during group allocation, data collection and/or data analysis

Please outline where this information can be found within the submission (e.g., sections or figure legends), or explain why this information doesn’t apply to your submission:

In our study all mice were randomly allocated into the different experimental groups.

All experimenters were blinded to the treatment conditions during image analysis.

This information is given in the section "Image processing and manual analysis".

**Additional data files (“source data”)**

- We encourage you to upload relevant additional data files, such as numerical data that are represented as a graph in a figure, or as a summary table
- Where provided, these should be in the most useful format, and they can be uploaded as “Source data” files linked to a main figure or table
- Include model definition files including the full list of parameters used
- Include code used for data analysis (e.g., R, MatLab)
- Avoid stating that data files are “available upon request”

Figure 3; Figure 4; Figure 5; Figure S7; Figure S9; Figure S10. The source codes are provided in a GitHub repository. Data and code are provided in a Dryad repository.

Please indicate the figures or tables for which source data files have been provided:
